# Using the Prevalence of Individual Species of Intestinal Nematode Worms to Estimate the Combined Prevalence of Any Species

**DOI:** 10.1371/journal.pntd.0000655

**Published:** 2010-04-13

**Authors:** Nilanthi de Silva, Andrew Hall

**Affiliations:** 1 Department of Parasitology, Faculty of Medicine, University of Kelaniya, Ragama, Sri Lanka; 2 Centre for Public Health Nutrition, University of Westminster, London, United Kingdom; McGill University Health Centre, Canada

## Abstract

**Objectives:**

To assess if a probabilistic model could be used to estimate the combined prevalence of infection with any species of intestinal nematode worm when only the separate prevalence of each species is reported, and to estimate the extent to which simply taking the highest individual species prevalence underestimates the combined prevalence.

**Methods:**

Data were extracted from community surveys that reported both the proportion infected with individual species and the combined proportion infected, for a minimum sample of 100 individuals. The predicted combined proportion infected was calculated based on the assumption that the probability of infection with one species was independent of infection with another species, so the probability of combined infections was multiplicative.

**Findings:**

Thirty-three reports describing 63 data sets from surveys conducted in 20 countries were identified. A strong correlation was found between the observed and predicted combined proportion infected (r = 0.996, *P*<0.001). When the observed and predicted values were plotted against each other, a small correction of the predicted combined prevalence by dividing by a factor of 1.06 achieved a near perfect correlation between the two sets of values. The difference between the single highest species prevalence and the observed combined prevalence was on average 7% or smaller at a prevalence of ≤40%, but at prevalences of 40–80%, the difference was about 12%.

**Conclusions:**

A simple probabilistic model of combined infection with a small correction factor is proposed as a novel method to estimate the number of individuals that would benefit from mass deworming when data are reported only for separate species.

## Author Summary

Mixed infections with roundworm, whipworm and hookworm are common, but survey reports often give only the separate prevalence of each type. However, the combined prevalence is important to estimate accurately the number of individuals who would benefit from control programmes and to make decisions about the frequency of treatment. Previous work suggests that mixed infections involving hookworm occur randomly, but that roundworm and whipworm infections are found together more frequently than would be expected by chance. We used 63 data sets from community surveys that reported both the proportions infected with individual types of worms and the combined proportion infected with any worm. We then calculated the proportion that would be infected with any type of worm if infections had occurred randomly and compared it with the observed combined proportion infected. We found a strong correlation between the observed and predicted combined proportions infected. A small downward correction of the predicted proportion infected by dividing by a factor of 1.06 brought it to a value that nearly equalled the observed proportion infected almost all the time. This simple model could be applied to published survey data to estimate accurately the number of individuals that would benefit from mass deworming.

## Introduction

The World Health Organization (WHO) estimates that intestinal nematode worms, also known as soil-transmitted helminths, are currently endemic in 130 countries in the world [1]. These worms include the common roundworm *Ascaris lumbricoides*, the whipworm *Trichuris trichiura* and the hookworms *Ancylostoma duodenale* and *Necator americanus*, which are usually treated as a single type as the eggs are indistinguishable under a microscope. In places where these worms are endemic, infections with two or three types, are commonly observed. Such mixed infections may occur randomly, as a simple probabilistic function of the prevalence of each individual species, or there may be factors that result in non-random association between species. The latter is possible, particularly because these worms are all transmitted on soil that has been contaminated with faeces from infected people. A probabilistic model to predict the prevalence of multi-species worm infections in human communities was proposed by Booth & Bundy in 1995 [2]. In testing this model against field data using log-linear analysis, it was found that combined infections with *A. lumbricoides* and *T. trichiura* occurred more frequently than expected by chance [2]. The authors concluded that their model was more effective in predicting the numbers of multiple infections involving hookworms than those involving only *A. lumbricoides* and *T. trichiura*.

As all these worms can be treated using a single dose of an inexpensive anthelmintic drug, the WHO recommends a strategy called “preventive chemotherapy” [3]. This involves annual mass treatment in all communities in which the prevalence of infection with any type of intestinal nematode worm among school-aged children is 20% or more, and twice yearly mass treatment if the prevalence is 50% or higher [3]. When mapping the prevalence of all intestinal nematode infections in order to determine the frequency of treatment, the WHO Global Databank on Schistosomiasis and Soil-Transmitted Helminths simply uses the highest prevalence [4] when surveys do not report the combined prevalence, and give only the separate prevalence of each species. This is done perhaps because the extent to which concurrent infections affect the accuracy of predictions made by the probabilistic model is not known.

With the resurgence of interest in controlling soil-transmitted helminth infections, much more field survey data are now available than when the probabilistic model was first proposed and tested [2]. The principal aim of the analysis reported here was to examine the accuracy of the probabilistic model in estimating the combined prevalence of intestinal nematode worm infection using data from a wide range of countries in all regions of the world, but using a simpler mathematical approach that could be easily applied. The subsidiary aims were to estimate the extent to which taking the highest individual prevalence underestimates the combined prevalence and to assess the degree of correlation between the proportions infected with each species of worm.

## Methods

A database of 230 publications in peer-reviewed journals, grey and unpublished literature that had been compiled in 2003 to estimate the global prevalence of intestinal nematode worms (described in ref. [5]) was searched for data that reported both the proportion infected with each species and the combined proportion infected. This was updated with a PubMed search limited to papers published in the last 10 years in English with free online access to the full text, using the terms ‘Soil transmitted helminths prevalence’ and ‘Ascaris AND Trichuris AND hookworm AND prevalence’. Only data from community-based studies with a sample size of >100 and published after 1990 were included. Where surveys had been carried out in several areas within a country and the results were presented in a geographically disaggregated manner, they were treated separately, rather than as a single data set. Data on prevalence is usually presented in the form of a percentage, with values ranging from 0 to 100. For purposes of this analysis, the percentage prevalences were converted into the proportion infected, with values ranging from 0 to 1.

**Figure 1:**
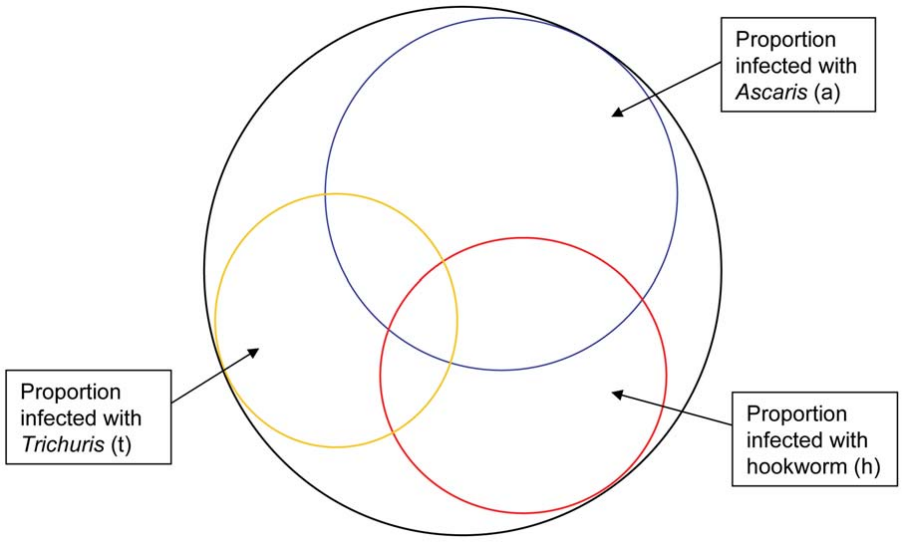
A diagrammatic representation of the permutation of infections with *Ascaris lumbricoides*, *Trichuris trichiura* and hookworm in a community in which all three occur.

Figure 1 represents the seven possible permutations of infections with *A. lumbricoides*, *T. trichiura* and the hookworms. The data from each survey were extracted as follows:

a = Proportion infected with *Ascaris*
t = Proportion infected with *Trichuris*
h = Proportion infected with hookworms

The proportions infected with each permutation of infection were then calculated as:
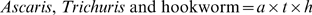


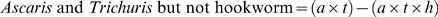


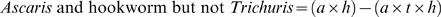


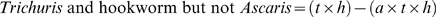


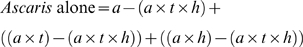


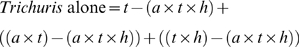


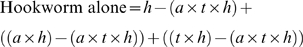

The combined proportion infected with *Ascaris*, *Trichuris* and hookworms (p_ath_) is thus the sum of all seven equations above:
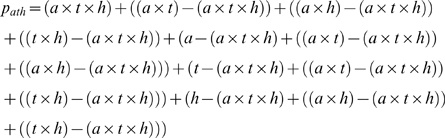

This can be simplified by cancellation to:
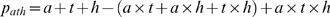
(1)
If only two species were present, such as *Ascaris* and *Trichuris*, then the proportion of double and single infections is calculated in a similar way:
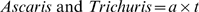


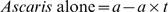


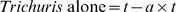

So the combined proportion infected with *Ascaris* and *Trichuris* (p_at_) is the sum of the three equations above: 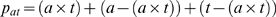.

This can be simplified by cancellation to: 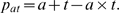
(2)The same simplified equations for infections with *Ascaris* and hookworm (*p_ah_*) and *Trichuris* and hookworm (*p_th_*) can be written as:
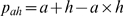
(3)

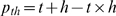
(4)


Equation 1 was applied in an Excel spreadsheet to calculate the predicted combined proportion infected from the data from each survey and the values were plotted against the observed combined proportion infected in the same survey. When only two worms were identified in a survey if a value of zero is entered for the missing type then the spreadsheet calculates the correct proportion infected with either or both species and it is not necessary to apply Equations 2 to 4.

To investigate the degree to which the highest single species prevalence may underestimate the combined prevalence, the differences between the highest individual species value and the observed combined proportion infected were plotted against the observed combined proportion infected.

To investigate the degree to which individual species were associated, correlation coefficients (r) were calculated for data derived from all surveys for the proportions infected with *Ascaris* and *Trichuris*, *Ascaris* and hookworm, and *Trichuris* and hookworm.

## Results

Thirty-three papers describing surveys conducted in 20 different countries were identified for this analysis: eight in Asia, six in Africa, five in Latin America and the Caribbean, and one in Oceania. Together they contained 63 sets of data: 30 from Asia, 23 from Africa, nine from Latin America & the Caribbean and one from Oceania (see Annex S1). The observed combined prevalences included in the analysis ranged from 1.9%, recorded in the Southern Highlands of Malawi, to 96.8%, recorded in Feni District in Bangladesh. Fifteen data sets (24%) had only two worm infections; the rest had three.

**Figure 2:**
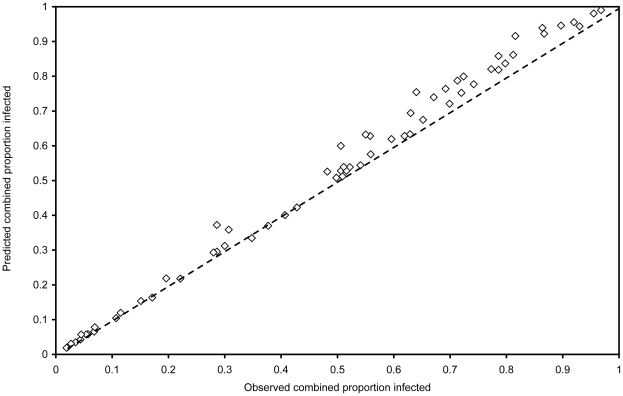
A scatter plot of the observed combined proportion infected with any intestinal nematode worms and the predicted combined prevalence.

**Figure 3:**
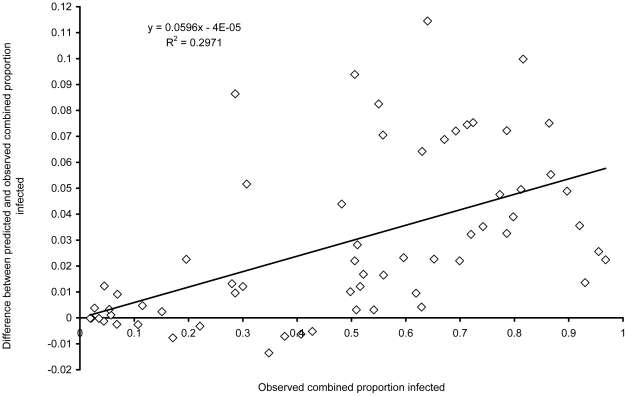
A scatter plot of the observed combined proportion infected with intestinal nematode worms and the difference between the predicted and observed proportion infected.

Figure 2 shows a scatter plot of the observed proportion infected against the predicted combined proportion infected (r = 0.996, *P*<0.001) with the line of equivalence. As the predicted combined proportion infected in Figure 2 tends to be above the line of equivalence, Figure 3 shows the observed combined proportion infected plotted on the x-axis, plotted against the difference between the observed and predicted proportions on the y-axis. The slope of the equation for the line in Figure 3 is 0.0596 rounded to 0.06, which indicates that the overestimation shown in Figure 2 increases by 0.06 for every 10% increase in prevalence. This provides a factor by which to correct the over-estimation of the predicted combined proportion infected (p_ath_) so that:
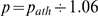
(5)


A plot of the observed combined proportion infected against this adjusted predicted combined proportion infected (not shown since it is almost identical to Figure 2) gave an equation for the line of 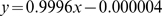, indicating a intercept of almost zero, a slope of almost 1 and correlation coefficient of r = 0.996.

**Figure 4:**
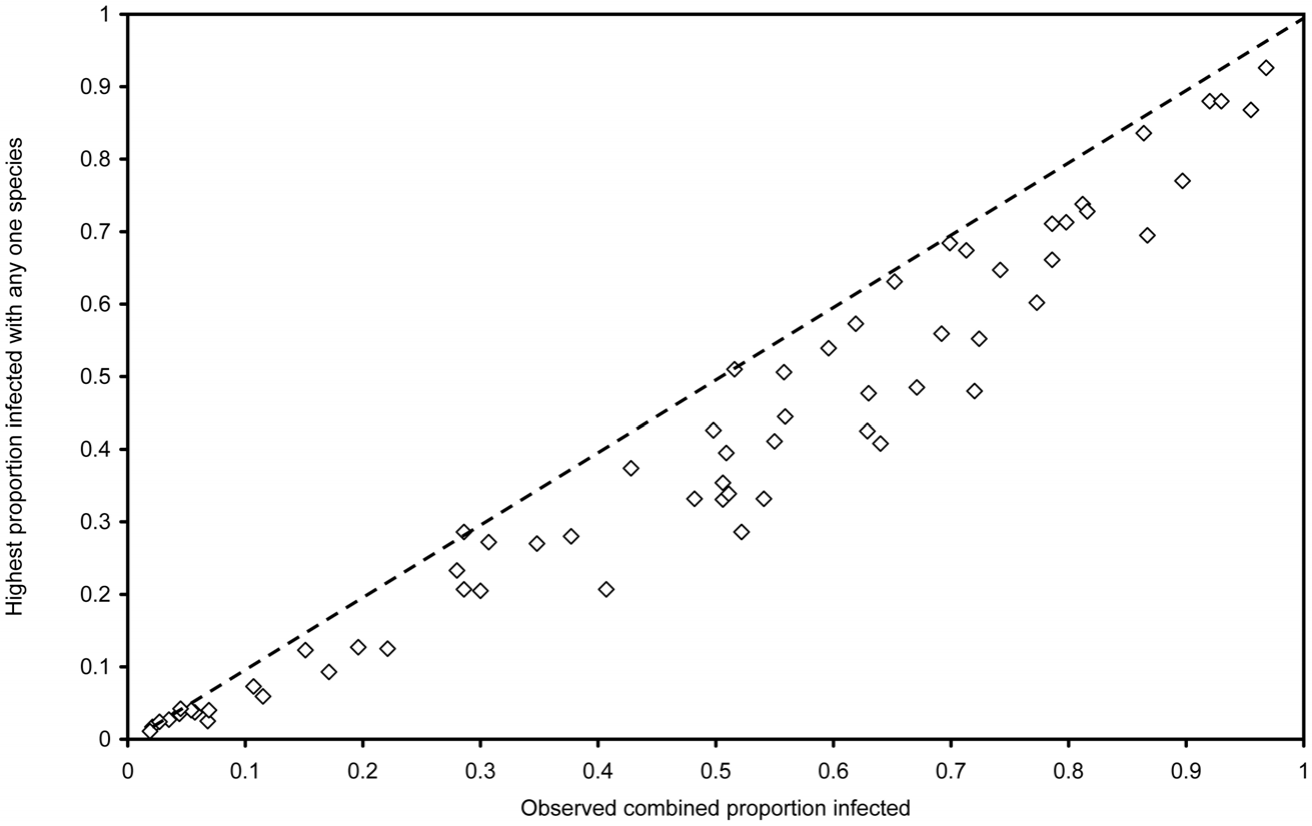
A scatter plot of the observed combined proportion infected with intestinal nematode worms against the proportion infected with the most common species.

Figure 4 shows a scatter plot of the observed combined proportion infected against the proportion infected with the single most common species, with the line of equivalence. It shows that the prevalence of the single most common species usually underestimates the combined prevalence, with the smallest differences occurring at the lowest and highest prevalences. The correlation coefficient of r = 0.973 was less than that between the observed and adjusted predicted combined proportion infected (0.996).

**Figure 5:**
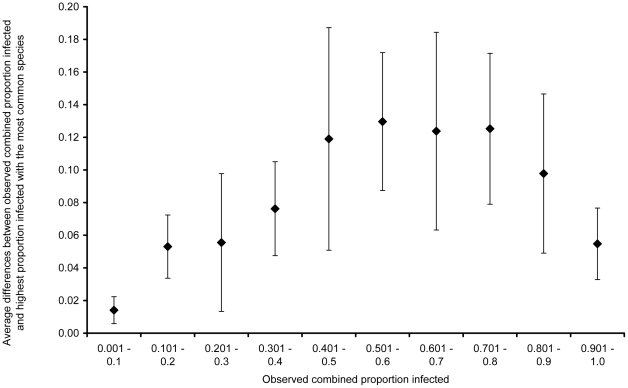
A plot of the average difference between the observed combined proportion infected and the proportion infected with the most common species for ten centiles of combined proportions infected in 63 surveys, with 95% confidence intervals.

To assess the magnitude of these underestimates in relation to the prevalence, Figure 5 shows a plot of the average difference between the observed combined proportion infected in the 63 data sets and the proportion infected with the single most common species for ten centiles of combined proportions infected. Between 3 and 11 data points were available to calculate the average for each centile. Figure 5 shows that when the observed combined proportion infected is 0.4–0.8 the observed combined prevalence is about 12% higher than the highest prevalence of any one species, with 95% CI ranging from about 6–18%.

The data were also analysed for correlations between proportions infected with *Ascaris* and *Trichuris*, *Ascaris* and hookworm, and *Trichuris* and hookworm in the 63 pairs of data points. The correlation coefficients were 0.544, 0.191 and 0.180 respectively, indicating a much stronger correlation between *Ascaris* and *Trichuris* than other pairs of infections. Seven of the 63 data sets also presented disaggregated data on the observed number of single, double and triple infections [6]–[11]. Of the seven, six were from communities that had both *Ascaris* and *Trichuris* infections; and in five of these, the observed prevalence of co-infection was higher than that expected to occur by chance.

## Discussion

This paper presents a simple equation (Eqn 1) to estimate the combined prevalence of infection with *A.lumbricoides*, *T.trichiura* and the hookworms from data on the separate prevalence of infection with each type. The combined prevalence can then be corrected (Eqn 5) to allow for the estimated degree of association between types, probably between *A.lumbricoides* and *T.trichiura*.

The strong correlation reported here between the observed and predicted combined prevalences supports the hypothesis proposed by Booth & Bundy [2] that when the three main species of intestinal nematode worms co-occur, the probability of infection with one species is largely independent of infection with another. This is despite the use of two different forms of analysis in testing the probabilistic model against field data. The results presented here also confirm the findings of Booth & Bundy that concurrent infections of *A. lumbricoides* and *T. trichiura* are more common than expected by chance. Several other studies have also noted this association [12]–[19], which probably arises from their common mode of transmission. One hypothesis argues that both these worms are transmitted in a “domestic” domain, within and around the house, while hookworm is transmitted in a public domain [20].

However, the present analysis also shows that a small downward correction of the predicted combined proportion infected is enough to achieve a very high correlation between predicted values and values reported by field surveys. The apparent over-estimation of combined prevalence probably results from the association between *A. lumbricoides* and *T. trichiura*. This over-estimation does not seem to be very large however, and is easily corrected. Equation 1, to estimate the combined proportion infected, and Equation 5, which provides a correction factor, could thus provide a novel and relatively simple and practical method to estimate the combined prevalence of infection with any intestinal nematode worm from data published on the prevalence of separate species.

This analysis does not take into account potential errors in parasitological diagnosis, particularly false negatives leading to an underestimated prevalence. The sensitivity and specificity of diagnosis are likely to be related to the concentration of eggs in faeces, which is related to fecundity of worms, the dispersal and dilution of eggs in the faecal mass, and to the amount of faeces examined under a microscope [21]. As these factors affect all three types of worms if present, they should not affect the analysis presented here, only that any combined prevalence could be an underestimate of the true prevalence of infection.

The difference between the prevalence of the single most common species of intestinal nematode, which is currently used by the WHO in the absence of data on combined prevalence, and the observed combined prevalence, seems to vary depending on the prevalence. At a combined prevalence of ≤40%, the difference is on average 7% or smaller, but when the combined prevalence is higher, the difference is about 12%. The difference is less also when the combined prevalence is very high (>90%). This has implications for mass treatment, especially at low prevalence rates. For example, if the proportion infected with *Ascaris* is 0.15, and the proportion infected with *Trichuris* is also 0.15, then the combined proportion infected is 0.2775 (0.15+0.15−0.0225). This prevalence of 28% is above the threshold at which the WHO currently recommends mass treatment, but the highest single species prevalence of 15% is below the threshold value of 20%. At higher prevalences the underestimation due to using of the highest single species prevalence is of less importance.

This analysis includes a modest number of data sets from all major geographical areas where intestinal nematode infections are endemic. It suggests that a simple probabilistic model with a small correction could be used to estimate the proportion of people infected with any intestinal nematode worm. This could help with the global mapping of disease and is likely to increase the estimated number of individuals that would benefit from mass deworming in the world today.

## Supporting Information

Annex S1 Data used for analysis.
(0.24 MB DOC)
