# Supplementary material for: Using the Prevalence of Individual Species of Intestinal Nematode Worms to Estimate the Combined Prevalence of Any Species
Source: PLoS Negl Trop Dis. 2010 Apr 13;4(4):e655. doi: 10.1371/journal.pntd.0000655 (PMC2854118; doi:10.1371/journal.pntd.0000655)
Supplement: Annex S1 — Data used for analysis. (0.24 MB DOC) [file pntd.0000655.s001.doc]

**Annex S1**

|  |  |  | **Proportion infected** | | | | | |  |
| --- | --- | --- | --- | --- | --- | --- | --- | --- | --- |
| **Continent/**  **Country** | **Community** | **Sample size** | ***Ascaris*** | ***Trichuris*** | **Hook**  **worm** | **Any worm (observed)** | **Any worm (expected)** | **Most common** | **Reference** |
| **AFRICA** |  |  |  |  |  |  |  |  |  |
| Ethiopia | Addis Ababa | 406 | 0.207 | 0.101 | 0.012 | 0.286 | 0.296 | 0.207 | 1 |
| Ethiopia | Afar | 367 | 0.035 | 0.003 | 0.005 | 0.044 | 0.048 | 0.035 | 1 |
| Ethiopia | Amhara | 1,195 | 0.280 | 0.055 | 0.074 | 0.377 | 0.370 | 0.280 | 1 |
| Ethiopia | B-Gumuz | 396 | 0.207 | 0.053 | 0.202 | 0.407 | 0.401 | 0.207 | 1 |
| Ethiopia | Dire Dawa | 403 | 0.037 | 0.017 | 0.005 | 0.057 | 0.058 | 0.037 | 1 |
| Ethiopia | Gambella | 393 | 0.331 | 0.242 | 0.211 | 0.506 | 0.600 | 0.331 | 1 |
| Ethiopia | Harari | 401 | 0.022 | 0.025 | 0.020 | 0.068 | 0.066 | 0.025 | 1 |
| Ethiopia | Oromiya | 1,575 | 0.205 | 0.046 | 0.093 | 0.300 | 0.312 | 0.205 | 1 |
| Ethiopia | SNNP | 1,148 | 0.395 | 0.099 | 0.105 | 0.509 | 0.512 | 0.395 | 1 |
| Ethiopia | Somali | 382 | 0.073 | 0.031 | 0.003 | 0.107 | 0.104 | 0.073 | 1 |
| Ethiopia | Tigray | 800 | 0.093 | 0.029 | 0.050 | 0.171 | 0.163 | 0.093 | 1 |
| Ethiopia | Gondar | 8006 | 0.354 | 0.127 | 0.163 | 0.506 | 0.528 | 0.354 | 2 |
| Kenya | Asembo | 1246 | 0.223 | 0.179 | 0.425 | 0.629 | 0.633 | 0.425 | 3 |
| Kenya | Bungoma | 574 | 0.056 | 0.009 | 0.059 | 0.115 | 0.120 | 0.059 | 4 |
| Malawi | C Highland | 242 | 0.004 | 0.000 | 0.017 | 0.021 | 0.021 | 0.017 | 5 |
| Malawi | N Highland | 257 | 0.008 | 0.000 | 0.027 | 0.035 | 0.035 | 0.027 | 5 |
| Malawi | S Highland | 261 | 0.008 | 0.000 | 0.011 | 0.019 | 0.019 | 0.019 | 5 |
| Malawi | S lowland | 297 | 0.007 | 0.000 | 0.024 | 0.027 | 0.031 | 0.024 | 5 |
| Malawi | Urban | 277 | 0.040 | 0.000 | 0.018 | 0.054 | 0.057 | 0.040 | 5 |
| S Africa | Cape Town | 3890 | 0.248 | 0.506 | 0.000 | 0.558 | 0.629 | 0.506 | 6 |
| Tanzania | Mafia Island | 201 | 0.030 | 0.353 | 0.711 | 0.786 | 0.819 | 0.711 | 7 |
| Tanzania | Mafia Island | 202 | 0.054 | 0.441 | 0.738 | 0.812 | 0.862 | 0.738 | 7 |
| Uganda | 18 districts | 2004 | 0.175 | 0.073 | 0.445 | 0.559 | 0.576 | 0.445 | 8 |

|  |  |  | **Proportion infected** | | | | | |  |
| --- | --- | --- | --- | --- | --- | --- | --- | --- | --- |
| **Continent/**  **Country** | **Community** | **Sample size** | ***Ascaris*** | ***Trichuris*** | **Hook**  **worm** | **Any worm (observed)** | **Any worm (expected)** | **Most common** | **Reference** |
| **ASIA** |  |  |  |  |  |  |  |  |  |
| Afghanistan | Farah | 249 | 0.426 | 0.129 | 0.000 | 0.498 | 0.508 | 0.426 | 9 |
| Afghanistan | Kabul | 239 | 0.573 | 0.130 | 0.000 | 0.619 | 0.629 | 0.573 | 9 |
| Afghanistan | Kandahar | 257 | 0.374 | 0.078 | 0.000 | 0.428 | 0.423 | 0.374 | 9 |
| Afghanistan | Nangahar | 256 | 0.270 | 0.063 | 0.027 | 0.348 | 0.335 | 0.270 | 9 |
| Bangladesh | Feni district | 285 | 0.825 | 0.926 | 0.256 | 0.968 | 0.990 | 0.926 | 10 |
| Bangladesh | Khagrachari | 250 | 0.408 | 0.360 | 0.352 | 0.640 | 0.755 | 0.408 | 10 |
| Bangladesh | Patuakhali | 257 | 0.580 | 0.661 | 0.004 | 0.786 | 0.858 | 0.661 | 10 |
| China | Lugao | 310 | 0.632 | 0.600 | 0.868 | 0.955 | 0.981 | 0.868 | 11 |
| China | Xinjian | 334 | 0.443 | 0.093 | 0.485 | 0.671 | 0.740 | 0.485 | 11 |
| China | national | 356629 | 0.127 | 0.046 | 0.061 | 0.196 | 0.219 | 0.127 | 12 |
| China | Jianmiao | 475 | 0.286 | 0.261 | 0.126 | 0.522 | 0.539 | 0.286 | 13 |
| China | Yaojiakon | 405 | 0.020 | 0.015 | 0.123 | 0.151 | 0.153 | 0.123 | 13 |
| China | Zhongzhou | 488 | 0.250 | 0.090 | 0.332 | 0.541 | 0.544 | 0.332 | 14 |
| China | Linger | 462 | 0.602 | 0.357 | 0.299 | 0.773 | 0.821 | 0.602 | 15 |
| China | Liuki | 304 | 0.434 | 0.158 | 0.480 | 0.720 | 0.752 | 0.480 | 15 |
| India | Villupuram | 646 | 0.539 | 0.057 | 0.124 | 0.596 | 0.619 | 0.539 | 16 |
| India | Quilon district | 996 | 0.713 | 0.371 | 0.097 | 0.798 | 0.837 | 0.713 | 17 |
| India | Visakhapatnam | 217 | 0.728 | 0.659 | 0.092 | 0.816 | 0.916 | 0.728 | 18 |
| Myanmar | Coastal | 250 | 0.576 | 0.836 | 0.124 | 0.864 | 0.939 | 0.836 | 19 |
| Myanmar | Delta | 250 | 0.630 | 0.880 | 0.000 | 0.920 | 0.956 | 0.880 | 19 |
| Myanmar | Hilly area | 250 | 0.233 | 0.032 | 0.048 | 0.280 | 0.293 | 0.233 | 19 |
| Myanmar | Plains | 250 | 0.500 | 0.552 | 0.104 | 0.724 | 0.799 | 0.552 | 19 |
| Nepal | Plains | 641 | 0.510 | 0.000 | 0.037 | 0.516 | 0.528 | 0.510 | 20 |
| Nepal | 3 districts | 426 | 0.273 | 0.041 | 0.339 | 0.511 | 0.539 | 0.339 | 21 |
| Nepal | 3 districts | 711 | 0.219 | 0.192 | 0.647 | 0.742 | 0.777 | 0.647 | 21 |
| N Korea | NK-China border | 282 | 0.411 | 0.376 | 0.000 | 0.550 | 0.633 | 0.411 | 22 |
| Sri Lanka | Ragama | 265 | 0.008 | 0.042 | 0.008 | 0.045 | 0.057 | 0.042 | 23 |
| Sri Lanka | National | 2162 | 0.028 | 0.040 | 0.012 | 0.069 | 0.078 | 0.040 | 24 |
| Sri Lanka | Plantations | 246 | 0.695 | 0.565 | 0.414 | 0.867 | 0.922 | 0.695 | 25 |
| Sri Lanka | Plantations | 1614 | 0.770 | 0.694 | 0.232 | 0.897 | 0.946 | 0.770 | 25 |

|  |  |  | **Proportion infected** | | | | | |  |
| --- | --- | --- | --- | --- | --- | --- | --- | --- | --- |
| **Continent/**  **Country** | **Community** | **Sample size** | ***Ascaris*** | ***Trichuris*** | **Hook**  **worm** | **Any worm (observed)** | **Any worm (expected)** | **Most common** | **Reference** |
| **LATIN AMERICA** | |  |  |  |  |  |  |  |  |
| Brazil | Amazonas State | 213 | 0.286 | 0.113 | 0.009 | 0.286 | 0.372 | 0.286 | 26 |
| Brazil | Amazonas State | 308 | 0.477 | 0.256 | 0.214 | 0.630 | 0.694 | 0.477 | 26 |
| Brazil | Ortigueira reserve | 100 | 0.880 | 0.020 | 0.520 | 0.930 | 0.944 | 0.880 | 27 |
| Brazil | Ortigueira urban | 136 | 0.125 | 0.051 | 0.058 | 0.221 | 0.218 | 0.125 | 27 |
| Ecuador | Portoviejo | 141 | 0.631 | 0.106 | 0.014 | 0.652 | 0.675 | 0.631 | 28 |
| Ecuador | Napo Province | 199 | 0.332 | 0.065 | 0.241 | 0.482 | 0.526 | 0.332 | 29 |
| Jamaica | Mandeville | 593 | 0.305 | 0.674 | 0.062 | 0.713 | 0.788 | 0.674 | 30 |
| Mexico | Coatzalcoalcos | 1384 | 0.465 | 0.559 | 0.000 | 0.692 | 0.764 | 0.559 | 31 |
| Panama | Cocle Provine | 658 | 0.000 | 0.272 | 0.119 | 0.307 | 0.359 | 0.272 | 32 |
|  |  |  |  |  |  |  |  |  |  |
| **OCEANIA** |  |  |  |  |  |  |  |  |  |
| Tuvalu | Nukutefau | 206 | 0.000 | 0.684 | 0.117 | 0.699 | 0.721 | 0.684 | 33 |

**References for Annex S1**

1. Hall A, Tamiru K, Tsegaye D, Tedbabe D, Seung Lee (2007) A national survey of the health of school children in Ethiopia*.* Addis Ababa: Save the Children, USA.
2. Jemaneh L, Lengeler C (2001). The use of morbidity questionnaires to identify communities with high prevalence of geohelminth infections in Gondar region, Ethiopia. Ethiop Med J 39: 213-228.
3. Handzel T, Karanja DM, Addiss DG, Hightower AW, Rosen DH, et al (2003) Geographic distribution of schistosomiasis and soil-transmitted helminths in Western Kenya: implications for anthelminthic mass treatment. Am J Trop Med Hyg 69: 318-23.
4. Centers for Disease Control and Prevention (2000) Palmar pallor as an indicator for anthelminthic treatment among ill children aged 2-4 years - Western Kenya, 1998. MMWR Morb Mortal Wkly Rep 49: 278-281.
5. Bowie C, Purcell B, Shaba B, Makaula P, Perez M (2004) A national survey of the prevalence of schistosomiasis and soil transmitted helminths in Malawi. BMC Infect Dis 16: 49.
6. Adams VJ, Markus MB, Adams JF, Jordaan E, Curtis B, et al (2005) Paradoxical helminthiasis and giardiasis in Cape Town, South Africa: epidemiology and control. Afr Health Sci 5: 131-136.
7. Albonico M, Ramsan M, Wright B, Jape K, Haji HJ, et al (2002) Soil-transmitted nematode infections and mebendazole treatment in Mafia Island schoolchildren. Ann Trop Med Parasitol 96: 717-726.
8. Kabatereine NB, Tukahebwa EM, Brooker S, Alderman H, Hall A (2001) The epidemiology of intestinal helminth infections among schoolchildren in 18 districts of southern Uganda. East Afr Med J 78: 283-286.
9. Gabrielli AF, Ramsan M, Naumann C, Tsogzolmaa D, Bojang B, et al (2005) Soil-transmitted helminths and haemoglobin status among Afghan children in World Food Programme assisted schools. J Helminthol 79: 381-384.
10. Hossain M, Allen H, Padmasiri EA, Bangali Am, Al Mamoon AB, et al (2005) Report on baseline survey on soil-transmitted helminths among school children. Dhaka: Directorate General of Health Service.
11. Liu CH, Zhang XR, Qiu DC, Xiao SH, Hotez PJ, et al (1999) Epidemiology of human hookworm infections among adult villages in Hejiang and Santai Counties, Sichuan Province, China. Acta Trop 73: 243-249.
12. Ministry of Health, Government of China (2005) Report on the national survey of current situation of major human parasite diseases in China. Beijing: National Institute of Parasitic Diseases and China CDC.
13. Sun FH, Wu ZX, Qian YX, Cao HQ, Xue HC, et al (1998) Epidemiology of human intestinal nematode infections in Wujiang and Pizhou Counties, Jiangsu Province, China. Southeast Asian J Trop Med Public Health 29: 605-610.
14. Wang Y, Shen GJ, Wu W, Xiao SH, Hotez PJ, et al (1999) Epidemiology of human ancylostomiasis among rural villages in Nanlin Country (Zhongzhou village) Anhui Province, China. Age associated prevalence, intensity and hookworm species identification. Southeast Asian J Trop Med Public Health 30: 692-697.
15. Zhang L, Zhang BX, Tao H, Xiao SH, Hotez P, et al (2000) Epidemiology of human geohelminth infections (ascariasis, trichuriasis, and necatoriasis) in Lushui and Puer Counties, Yunnan Province, China. Southeast Asian J Trop Med Public Health 31: 448-453.
16. Mani TR, Rajendran R, Munirathinam A, Sunish IP, Abdullah SM, et al (2002) Efficacy of co-administration of albendazole and diethylcarbamazine against geohelminthiases: a study from South India. Trop Med Int Health 7: 541-548.
17. Nair TNP (1991) Prevalence of intestinal helminthiasis and associated anaemia among pre-school children in a fishermen community in Kerala. Ann Natl Acad Med Sci 27: 129-134.
18. Paul I, Gnanamani G, Nallam NR (1999) Intestinal helminth infections among school children in Visakhapatnam. Indian J Pediatr 66: 669-673.
19. Montresor A, Thet Thet Zin, Padmasiri E, Allen A, Savioli L (2004). Soil-transmitted helminthiasis in Myanmar and approximate costs for country-wide control. Trop Med Int Health 9: 1012-1015.
20. Curtale F, Tilden R, Muhilal, Vaidya Y, Pokhrel RP, et al (1993) Intestinal helminths and risk of anaemia among Nepalese children. Panminerva Med 35: 159-166.
21. Montresor A, Celletti F (2000) Results of the school survey on soil-transmitted nematode infection and nutrition conducted in Parsa, Dadeldhura and Doti. Unpublished WHO report.
22. Li S, Shen C, Choi MH, Bae YM, Yoon H, et al (2006) Status of intestinal helminthic infections of borderline residents in North Korea. Korean J Parasitol 44: 265-268.
23. de Silva NR, Pathmeswaran A, Fernando SD, Weerasinghe CR, Selvaratnam R, et al (2003) Impact of mass chemotherapy for the control of filariasis on geohelminth infections in Sri Lanka. Ann Trop Med Parasitol 97: 421-425.
24. Pathmeswaran A, Jayatissa R, Samarasinghe S, Fernando A, de Silva RP, et al (2005) Health status of primary schoolchildren in Sri Lanka. Ceylon Med J 50: 46-50.
25. Sorensen E, Ismail M, Amarasinghe DKC, Hettiarachchi I, Dassenaieke TSdeC (1996) The prevalence and control of soil-transmitted nematode infections among children and women in the plantations in Sri Lanka. Ceylon Med J 41: 37-41.
26. Boia NM, Carvalho-Costa FA, Sodre FC, Eyer-Silva WA, Lamas CC, et al (2006) Mass treatment for intestinal helminthiasis control in an Amazonian endemic area in Brazil. Rev Inst Med Trop Sao Paulo 48: 189-195.
27. Scolari C, Torti C, Beltrame A, Matteelli A, Castelli F, et al (2000) Prevalence and distribution of soil-transmitted helminth (STH) infections in urban and indigenous schoolchildren in Ortigueira, State of Paranà, Brasil: implications for control. Trop Med Int Health 5: 302-307.
28. Andrade C, Alava T, De Palacio IA, Del Poggio P, Jamoletti C, et al (2001) Prevalence and intensity of soil-transmitted helminthiasis in the city of Portoviejo (Ecuador). Mem Inst Oswaldo Cruz 96:1075-1079.
29. San Sebastian M, Santi S (2000) Control of intestinal helminths in schoolchildren in Low-Napo, Ecuador: impact of a two-year chemotherapy program. Rev Soc Bras Med Trop 33: 69-73.
30. Nokes C, Cooper ES, Robinson BA, Bundy DAP (1991) Geohelminth infection and academic assessment in Jamaican children. Trans R Soc Trop Med Hyg 85: 272-273.
31. Forrester JE, Bailar III JC, Esrey SA, Jose MV, Castillejos BT, et al (1998) Randomised trial of albendazole and pyrantel in symptomless trichuriasis in children. Lancet 352: 1103-1108.
32. Robertson LJ, Crompton DW, Sanjur D, Nesheim MC (1992) *Trichuris trichiura* and the growth of primary schoolchildren in Panama. Trans R Soc Trop Med Hyg 86: 656-657.

33. Speare R, Latasi FF, Nelesone T, Harmen S, Melrose W, et al (2006) Prevalence of soil transmitted nematodes on Nukufetau, a remote Pacific island in Tuvalu. BMC Infect Dis 12:110.
